# Host response to influenza infections in human blood: association of influenza severity with host genetics and transcriptomic response

**DOI:** 10.3389/fimmu.2024.1385362

**Published:** 2024-08-13

**Authors:** Klaus Schughart, Amber M. Smith, Ephraim L. Tsalik, Stephen C. Threlkeld, Subhashini Sellers, William A. Fischer, Jens Schreiber, Eva Lücke, Markus Cornberg, Jennifer Debarry, Christopher W. Woods, Micah T. McClain, Mark Heise

**Affiliations:** ^1^ Institute of Virology Münster, University of Münster, Münster, Germany; ^2^ Department of Microbiology, Immunology and Biochemistry, University of Tennessee Health Science Center, Memphis, TN, United States; ^3^ Department of Pediatrics, University of Tennessee Health Science Center, Memphis, TN, United States; ^4^ Division of Infectious Diseases, Department of Medicine, Duke University School of Medicine, Durham, NC, United States; ^5^ Baptist Memorial Hospital, Memphis, TN, United States; ^6^ Division of Pulmonary Diseases and Critical Care Medicine, Department of Medicine, University of North Carolina at Chapel Hill, Chapel Hill, NC, United States; ^7^ Clinic of Pneumology, Otto-von-Guerike University, Magdeburg, Germany; ^8^ Centre for Individualised Infection Medicine (CiiM), a Joint Initiative of the Helmholtz Centre for Infection Research (HZI) and Hannover Medical School (MHH), Hannover, Germany; ^9^ Department of Gastroenterology, Hepatology, Infectious Diseases and Endocrinology, Hannover Medical School (MHH), Hannover, Germany; ^10^ German Centre for Infection Research (DZIF), Partner Site Hannover-Braunschweig, Hannover, Germany; ^11^ TWINCORE Centre for Experimental and Clinical Infection Research, a Joint Venture Between the Helmholtz-Centre for Infection Research (HZI) and the Hannover Medical School (MHH), Hannover, Germany; ^12^ Center for Infectious Disease Diagnostics and Innovation, Department of Medicine, Duke University School of Medicine, Durham, NC, United States; ^13^ Department of Genetics, University of North Carolina at Chapel Hill, Chapel Hill, NC, United States; ^14^ Department of Microbiology and Immunology, University of North Carolina at Chapel Hill, Chapel Hill, NC, United States

**Keywords:** influenza, human, transcriptome, genotype, DEGs, QTLs

## Abstract

**Introduction:**

Influenza virus infections are a major global health problem. Influenza can result in mild/moderate disease or progress to more severe disease, leading to high morbidity and mortality. Severity is thought to be primarily driven by immunopathology, but predicting which individuals are at a higher risk of being hospitalized warrants investigation into host genetics and the molecular signatures of the host response during influenza infections.

**Methods:**

Here, we performed transcriptome and genotype analysis in healthy controls and patients exhibiting mild/moderate or severe influenza (ICU patients). A unique aspect of our study was the genotyping of all participants, which allowed us to assign ethnicities based on genetic variation and assess whether the variation was correlated with expression levels.

**Results:**

We identified 169 differentially expressed genes and related molecular pathways between patients in the ICU and those who were not in the ICU. The transcriptome/genotype association analysis identified 871 genes associated to a genetic variant and 39 genes distinct between African-Americans and Caucasians. We also investigated the effects of age and sex and found only a few discernible gene effects in our cohort.

**Discussion:**

Together, our results highlight select risk factors that may contribute to an increased risk of ICU admission for influenza-infected patients. This should help to develop better diagnostic tools based on molecular signatures, in addition to a better understanding of the biological processes in the host response to influenza.

## Introduction

Influenza virus infections represent a major global health problem. High morbidity and mortality are observed, with up to 500,000 deaths each year worldwide (1) and millions during past pandemics (2, 3). Influenza infections cause a range of disease phenotypes that range from asymptomatic to severe. Severity is influenced by a variety of viral and host factors, including influenza strain, age, sex, host genetics, and immune status (*e.g.* (4–8). Mortality in severe cases is primarily driven by a pathological immune response characterized by high levels of neutrophils, macrophages, and inflammatory cytokines (7, 9–13). In addition, ethnicity appears to affect the severity of influenza and SARS-CoV-2 infections in the US. During the 2009 H1N1 pandemic rates of hospitalization were highest in African-Americans, observed in a study in Wisconsin (14). During the SARS-CoV-2 pandemic, African-Americans exhibited higher severity than Caucasians (15, 16).

To advance the diagnosis and better predict the probability of progression to severe influenza and risk of ICU admission, it is pivotal to characterize host responses in-depth. While obtaining samples directly from pulmonary infection sites presents challenges, the analysis of blood samples has emerged as a practical means to comprehensively assess the molecular aspects of the disease across the entire system. In addition, exploring whole-blood gene expression provides a valuable avenue for developing clinical tests for point-of-care use, enabling a more personalized and effective approach to patient management. Several transcriptome studies on samples from influenza-infected patients have been performed, with most using gene expression arrays (17–33). Some of these studies distinguished mild/moderate infections from severe influenza disease. For example, Bermejo-Martin et al. (19) observed impaired expression of several genes participating in the T cell and B cell immune responses in patients with severe influenza (patients requiring mechanical ventilation) compared to patients with reduced severity. These included genes involved in antigen presentation, B cell development, T helper cell differentiation, *CD28*, granzyme B signaling, apoptosis, and protein ubiquitination. Patients with the poorest outcomes were characterized by proinflammatory hypercytokinemia. In contrast, Dunning at al. (30) found an inflammatory, activated neutrophil, and cell stress or cell death pattern in patients who needed mechanical ventilation. Similarly, Tang et al. (32) observed elevated gene expression related to neutrophil activation in severe patients as the most important difference when compared to patients with moderate disease.

Because some identified genes may be cohort-specific and potentially a consequence of age, sex, and/or ethnicity, we aimed to identify the differences in gene expression among influenza-infected patients by taking these factors into account. We collected blood samples from a large cohort of influenza-infected patients from different parts of the US and Germany with severe or mild/moderate disease and healthy controls. We performed RNAseq and genotyping analyses on these samples. To our knowledge, this is the only study so far that performed gene expression analysis and genotyping on the same patients. This approach allowed us to assign ethnicity based on genotyping rather than skin phenotype or questionnaires. In addition, we were able to examine correlations between genetic variation and gene expression changes. The results suggested that while many differentially expressed genes (DEGs) and related molecular pathways in influenza-infected individuals were distinct from healthy controls, much less distinguished those who required ICU admission from non-ICU patients and very few were different for age, sex, or ethnic background.

## Materials and methods

### Patient cohorts – sample collections

Patients with influenza infections and healthy controls were collected at five different sites (**Data file 3**, see **Table 1** for availability of supplementary data and tables). Baptist Memorial Hospital (Memphis, TN USA): Patients with influenza virus infection confirmed by rapid-antigen assay/viral PCR performed on nasopharyngeal samples were recruited at admission to the hospital or from patients in the ICU at the time of consent (assigned as day 1). Blood samples from healthy controls were taken from hospital visitors and hospital patients with no respiratory infections. Otto-von-Gericke University (Magdeburg, Germany): Patients with influenza virus infection confirmed by rapid-antigen assay/viral PCR performed on nasopharyngeal samples were recruited at admission to the hospital. Blood samples were collected at admission (day 1). From ICU patients, samples were taken at the time of consent (the first sample taken was assigned as day 1). Blood samples from healthy controls were taken from hospital visitors, hospital patients with no respiratory or other infections or inflammatory disorders, and volunteers at the Helmholtz Centre for Infection Research, Braunschweig. Hannover Medical School (Hannover, Germany): Blood samples from healthy controls were taken from hospital patients without respiratory infections and volunteers at Hannover Medical School. University of North Carolina (Chapel Hill, NC USA): Patients with influenza virus infection confirmed by rapid-antigen assay or viral PCR performed on nasopharyngeal samples were recruited during hospital admission. Blood samples were collected at admission or from patients in the ICU at the time of consent (the first sample taken was assigned as day 1). Duke University (Durham, NC USA): Patients were enrolled by convenience sampling in the emergency department based on the presence of a suspected infection of less than 28 days duration. Participants were selected for inclusion in this study based on a diagnosis of influenza, which was based on PCR testing of nasopharyngeal swabs using the ResPlex V2.0 (Qiagen; Hilden, Germany), Respiratory Viral Panel (Luminex; Austin, TX), or Respiratory Pathogen Panel (Luminex; Austin, TX). Clinical adjudications were performed to confirm influenza was the microbiological etiology of illness, as previously described (28). See **Data file 5** for information on collection sites. A few samples were taken from a repository (seasons 2008/2009 until 2013/2014, total of 5 samples, collected at the Otto-von-Gericke University Magdeburg, Germany). Most samples were collected during subsequent seasons 2014/2015 until 2020/2010 (total of 208 samples; **Data file 4** for each season).

**Table 1:** Overview supplementary material at online repositories.

Label	Name of data file/data set	File types (file extension)	Data repository and identifier (DOI or accession number)
*Data file 1*	*Data set: supplements* *File: ST1_transcriptome_sbst1_target_comb_SIG_2018_2019_2020_2022_181122_2a.txt*	*txt*	*figshare https://doi.org/10.6084/m9.figshare.24299152*
*Data file 2*	*Data set: supplements* *File: ST2_coriell_samples_description_kls_310823.xlsx*	*Excel*	*figshare https://doi.org/10.6084/m9.figshare.24299152*
*Data file 3*	*Data set: supplements* *File: ST3_genotype_target_combined_unique_SIG_geno_assethn_310823a.txt*	*txt*	*figshare https://doi.org/10.6084/m9.figshare.24299152*
*Data file 4*	*Data set: supplements* *File: ST4_season_110823.xlsx*	*Excel*	*figshare https://doi.org/10.6084/m9.figshare.24299152*
*Data file 5*	*Data set: supplements* *File: ST5_Collection_site_040923.xlsx*	*Excel*	*figshare https://doi.org/10.6084/m9.figshare.24299152*
*Data file 6*	*Data set: supplements* *ST6_sbst1_limma_healthy_control_vs_infected_181122.txt*	*txt*	*figshare https://doi.org/10.6084/m9.figshare.24299152*
*Data file 7*	*Data set: supplements* *File: ST7_sbst1_limma_ICUn_vsCNRTL_181122.txt*	*txt*	*figshare https://doi.org/10.6084/m9.figshare.24299152*
*Data file 8*	*Data set: supplements* *File: ST8_sbst1_limma_ICUy_vsCNRTL_181122.txt*	*txt*	*figshare https://doi.org/10.6084/m9.figshare.24299152*
*Data file 9*	*Data set: supplements* *File: ST9_sbst1_limma_ICUy_vs_ICUn_181122.txt*	*txt*	*figshare https://doi.org/10.6084/m9.figshare.24299152*
*Data file 10*	*Data set: supplements* *File: ST10_sbst1_limma_ICUn_fVSm_231122.txt*	*txt*	*figshare https://doi.org/10.6084/m9.figshare.24299152*
*Data file 11*	*Data set: supplements* *File: ST11_sbst1_limma_ICUy_fVSm_231122.txt*	*txt*	*figshare https://doi.org/10.6084/m9.figshare.24299152*
*Data file 12*	*Data set: supplements* *File: ST12_sbst1_limma_old_young_060923.txt*	*txt*	*figshare https://doi.org/10.6084/m9.figshare.24299152*
*Data file 13*	*Data set: supplements* *File: ST13_cis_eQTL_DEGs_dist1Mb_result_011222_150923.txt*	*txt*	*figshare https://doi.org/10.6084/m9.figshare.24299152*
*Data file 14*	*Data set: supplements* *File: ST14_DEGs_CaucVSAfrAM_ciseQTL_121023.txt*	*txt*	*figshare https://doi.org/10.6084/m9.figshare.24299152*
*Data set 15*	*Data set: SIG_geno_0718*	*PLINK, txt*	*figshare https://doi.org/10.6084/m9.figshare.24299380*
*Data set 16*	*Data set: SIG_geno_0419*	*PLINK, txt*	*figshare https://doi.org/10.6084/m9.figshare.24297946*
*Data set 17*	*Data set: SIG_geno_2020*	*PLINK, txt*	*figshare https://doi.org/10.6084/m9.figshare.24299389*
*Data set 18*	*Data set: SIG_geno_2022*	*PLINK, txt*	*figshare https://doi.org/10.6084/m9.figshare.24299401*
*Data set 19*	*Data set: SIG_geno_combine*	*PLINK, txt*	*figshare https://doi.org/10.6084/m9.figshare.24299443*
*Data file 20*	*Data set: supplements* *File: ST20_sbst1_norm_LIMMA_btch_corr_SIG_comb_2022_2020_2019_2018_181122_1.txt*	*txt*	*figshare https://doi.org/10.6084/m9.figshare.24299152*
*Data file 21*	*Data set: supplements* *File: ST21_comb_numeric_genotypes_SIG_geno_ptntID_011222.7z*	*txt*	*figshare https://doi.org/10.6084/m9.figshare.24299152*
*Data set 22*	*SIG_2018*	*fastq*	*Sequence Read Archive: SRP285410* *GEO-ID: GSE158592*
*Data set 23*	*SIG2019*	*fastq*	*Sequence Read Archive: SRP275678* *GEO-ID: GSE155635*
*Data set 24*	*SIG_2020*	*fastq*	*GEO-ID: GSE196350*
*Data set 25*	*SIG_2022*	*fastq*	*GEO-ID: GSE213168*
*Data file 26*	*Data set: supplements* *File: ST26_gg4_geno_011222.7z*	*txt*	*figshare https://doi.org/10.6084/m9.figshare.24299152*
*Data file 27*	*Data set: supplements* *File: ST27_tr7_transcriptome_011222.txt*	*txt*	*figshare https://doi.org/10.6084/m9.figshare.24299152*
*Data file 28*	*Data set: supplements* *File: ST28_sbst1_limma_infAfrAm_vs_infCaucs_181122.txt*	*txt*	*figshare https://doi.org/10.6084/m9.figshare.24299152*
*Data file 29*	*ST29_ifn_old_071123.txt*	*txt*	*figshare https://doi.org/10.6084/m9.figshare.24299152*
*Data file 30*	*ST30_ifn_young_071123.txt*	*txt*	*figshare https://doi.org/10.6084/m9.figshare.24299152*

Data files and data sets generated or used in this study and their accessibility in public repositories.

### General aspects of sample analysis

The samples were analyzed in four different batches, although the preferred procedure would have been to process all samples in one batch. Analysis in different batches was mainly due to funding limitations. The grant support was provided in yearly parts, and therefore all samples from one season had to be analyzed within the corresponding fiscal year. Therefore, we provide the raw data from each batch, together with the combined data from all batches and the processed batch-corrected data files (see **Table 1** for availability of supplementary data and tables). Some samples were analyzed in multiple batches. In addition, for some patients, samples at multiple time points were collected. The analysis of the data presented here was performed with a unique sample from each participant and only those collected on day 1, when patients presented at the hospital, or the first blood draw from ICU patients. Therefore, in addition to the analyses presented here, our data may also be used to study gene expression changes in a single patient over time.

### Preparation of RNA and RNA sequencing

RNA was prepared from whole blood collected into PAXgene Blood RNA tubes (Qiagen) and then extracted as per the manufacturer’s protocol (QIAGEN PreAnalytiX – Blood RNA Kit). The quality and integrity of total RNA were controlled on an Agilent Technologies 2100 Bioanalyzer (Agilent Technologies; Waldbronn, Germany). Globin mRNA was depleted from total RNA using GLOBINclear Kit, human (ThermoFisher, Invitrogen). After globin mRNA depletion, the strand-specific RNA sequencing library was generated using the NEBNext Ultra II Directional RNA Library Prep Kit (New England Biolabs) according to the manufacturer’s protocols. The library was sequenced on Illumina HiSeq 4000 using the HiSeq 3000/4000 SBS Kit (300 cycles).

### Bioinformatics of RNAseq data

Reads were quality checked with package FastQC (version 0.11.4) (34), then trimmed using Trimgalore (version 0.4.4, (35)) with default settings. Trimmed reads were mapped to human genome annotation GRCh38 (ENSMBL Homo_sapiens.GRCh38.91) using STAR (version 2.5.2b, (36)) with default settings. Mapped reads were counted using RsubRead (version 1.32.4, (37)). Analysis and visualization of expression data was performed using the R software package (version 4.2.1, (38) and RStudio (version 2022.07.2, (39)). Annotation of human genes was performed using package biomaRt (version 2.52.0, annotation GRCh38.p12, (40)). Raw counts were then normalized using DESeq2 (version 1.16.1, (41)). The four transcriptome batches were: SIG_2018 (Data set 22), SIG2019 (Data set 23), SIG_2020 (Data set 24), and SIG_2022 (Data set 25). The respective raw and normalized data are available at the GEO (GEO) public database (see **Table 1** for availability of supplementary data and tables). The mean number of reads per batch were: 50 million, 45 million, 85 million, and 54 million, respectively. The normalized expression levels from all batches were then combined and batch-corrected using the Limma package (version 3.42.2; 42, 43)). The four batches contained overlapping samples analyzed in multiple batches. For subsequent analyses performed here, multiple samples from the same participant or reference were removed, and a unique dataset was generated (**Data file 1** for sample description and **Data file 20** for normalized batch corrected expression values, see **Table 1** for availability of supplementary data and tables). For the identification of differentially expressed genes (DEGs), the Limma package (version 3.42.2, (42, 43)) was used. The model used for the identification of DEGs in Limma was: design <- model.matrix(~ 0 + group), including all groups in the model. DEGs were determined by contrasting the groups from the Limma result, based on an adjusted p-value of < 0.05 and exhibiting more than a 1.5-fold (log_2_ = 0.5849625) difference in expression levels. Multiple testing adjusted *P* values were calculated according to Benjamini and Hochberg (44). Volcano plots were generated with the package EnhancedVolcano, version 1.8.0 (45). VENN diagrams were generated with the function vennPlot (http://faculty.ucr.edu/~tgirke/Documents/R_BioCond/My_R_Scripts/overLapper.R). Functional analysis of DEGs was performed using the R software package clusterProfiler (version v3.14.3; (46). For beeswarm graphs of expression levels, package beeswarm (47) (version 0.2.3.) was used.

### Preparation of DNA for genotyping

EDTA blood samples were collected from participants, cells were centrifuged, and supernatants and pellets were stored at -80 ° C. DNA was prepared from frozen cell pellets using the QIAamp DNA Blood Midi kit (Qiagen) according to the manufacturer’s protocol. For identification of ethnicity, DNA samples from the Coriell Institute HAPMAP collection were included as references. The biospecimens for the reference samples were donated by different populations (Population Descriptors, see **Data file 2**, see **Table 1** for availability of supplementary data and tables) and obtained from the NHGRI Sample Repository for Human Genetic Research at the Coriell Institute for Medical Research (Repository IDs see **Data file 2**, see **Table 1** for availability of supplementary data and tables). Here, we refer to the affiliation of a participant to their population as ethnicity (alternatively, race or genetic descent are used by others).

### Genotyping of DNA by SNP microarrays

Per sample, 2.5 µg of DNA was prepared for microarray analysis on Illumina Global Screening Array-24 v2.0 (Illumina), and DNA array analysis was performed according to the manufacturer’s protocol (Illumina, Infinium HTS Assay Manual Workflow) at the Johns Hopkins University School of Medicine, Genetic Resources Core Facility (GRCF). After QC, signals showing obvious assay failures on the array were removed. SNP calling was done with GenomeStudio version 2011.1, Genotyping Module version 1.9.4, and GenTrain Version 1.0. In total, 665,608 SNPs were probed on the genotyping arrays. The four genotyping batches were: SIG_geno_0718 (Data set 15), SIG_geno_0419 (Data set 16), SIG_geno_2020 (Data set 17), and SIG_geno_2022 (Data set 18). These datasets contain internal duplicates, duplicates between batches, and reference genomes. Several samples were analyzed in multiple batches, these duplicates were removed and a unique sample dataset was generated (**Data file 3** for description of the samples). The corresponding data are available as PLINK files (see **Table 1** for availability of supplementary data and tables).

### Bioinformatic analysis of genotype data

The combined genotype (SIG geno combined) data from all batches, a total of 365 samples (including all duplicates), were then analyzed by PLINK ( (48), see Data set 19 for PLINK and subjects). The respective PLINK file for the combined dataset (SIG_geno_combine) is provided in Data set 19 (see **Table 1** for availability of supplementary data and tables). Participant’s ethnicity was identified by multidimensional scaling (MDS) with reference to samples from the Coriell Institute HAPMAP collection (see results below). Numeric genotype calls were extracted from the four individual GenomeStudio batch files using the software GenomeStudio (Version 2.0) (49) and then combined into a single dataset. Subsequently, multiple samples from the same participant or the reference samples were removed, and a unique dataset was generated (**Data file 21** for the genotype data, **Data file 3** for a description of this sample set, see **Table 1** for availability of supplementary data and tables). Visualization and analyses of PLINK and GenomeStudio output files were performed using the R software package (50).

### QTL analysis

Overlapping datasets from the same participants for genotypes (numeric allele call data) and transcriptomes were generated. The corresponding genotype table is provided in **Data file 26**, and the corresponding transcriptome table is in **Data file 27** (see **Table 1** for availability of supplementary data and tables). QTL analysis was then performed using the R package MatrixEQTL (version 2.3) (51). Manhattan plots were generated using package qqman_0.1.8 (version 0.1.8; (52).

### Statistics

For the comparison of two groups, a two-way t-test (numeric data) or chi-square test (categorical data) was used and performed in R. P < 0.05 was considered significant. Multiple testing adjusted *P* values were calculated according to Benjamini and Hochberg (44).

### Availability of data and materials

All data are available in public databases. Raw data for RNAseq analyses are available in the GEO expression database (53–55): IDs: SIG_2018: GSE158592; SIG2019: GSE155635; SIG_2020: GSE196350; SIG_2022: GSE213168. Genotype raw data files are available in PLINK format at the Figshare public database (56), see **Table 1** for availability of supplementary data and tables. All other data files are available in Figshare (see **Table 1** for availability of supplementary data and tables).

## Results

### Cohort demographics

An overview of the demographics of patients and the different groups can be found in **Table 2**. The total number of participants was 208, 81 were healthy controls and 127 were influenza-infected patients, of which 23 were admitted to the ICU (**Table 2**). The genotyping analysis (see below) showed that the samples were primarily Caucasian (153) followed by African-American (people of African ancestry in the US, 41), Mexican-American-Indian (4), Asian (2), and admixed (2) (**Table 2**). The number of females and males was significantly different between infected and healthy controls (**Table 2**), but it was not significant between females and males when stratified by ICU status. The median age was significantly different between infected and control samples, it was not significantly different between non-ICU and ICU samples (**Table 2**). For details on each participant, see **Data file 1**.

**Table 2:** Demographics of cohorts.

Category	Healthy controls	Infected	Infected - not ICU	Infected - ICU	p-values HC versus infected	p-values non-ICU versus ICU
Gender (males/females)	59F/22M	66F/61M			< 0.01	
*sum*	*81*	*127*				
Gender (males/females)			54F/50M	12F/11M		1
sum			104	23		
AGE						
Age/years (median)	44	58	51	54	< 0.01	0.4536
Age/years (IQR)	(31 -76)	(45 - 68)	(37 - 62)	(38.5 - 68)		
Age range 18-30	10F/7M	7F/2M	6F/1M	1F/1M		
Age range 30-65	47F/14M	40F/42M	33F/36M	7F/6M		
Age range > 65	2F/1M	19F/17M	15F/13M	4F/4M		0.899
Ethnicity						
Caucasian	50F/15M	47F/41M	38F/32M	9F/9M		
*sum*	*65*	*88*	*70*	*18*		
African-American	7F/0M	18F/16M	15F/16M	3F/0M		
*sum*	*7*	*34*	*31*	*3*		
Asian	1F/1M	0	0	0		
Mexican American-Indian	0F/4M	0	0	0		
Admixed	0	1F/1M	1F/1M	0		

Number of participants stratified by gender, age, infection (infected: influenza infected, HC: healthy controls), and severity of disease (ICU: intensive care unit patients, non-ICU: patients not in intensive care). F: female, M: male. P-values were calculated by two-sided paired-t-test. Ethnicity was determined by genotype analysis (this study).

### Transcriptome analysis of infected patients versus healthy controls

**Figure 1:**
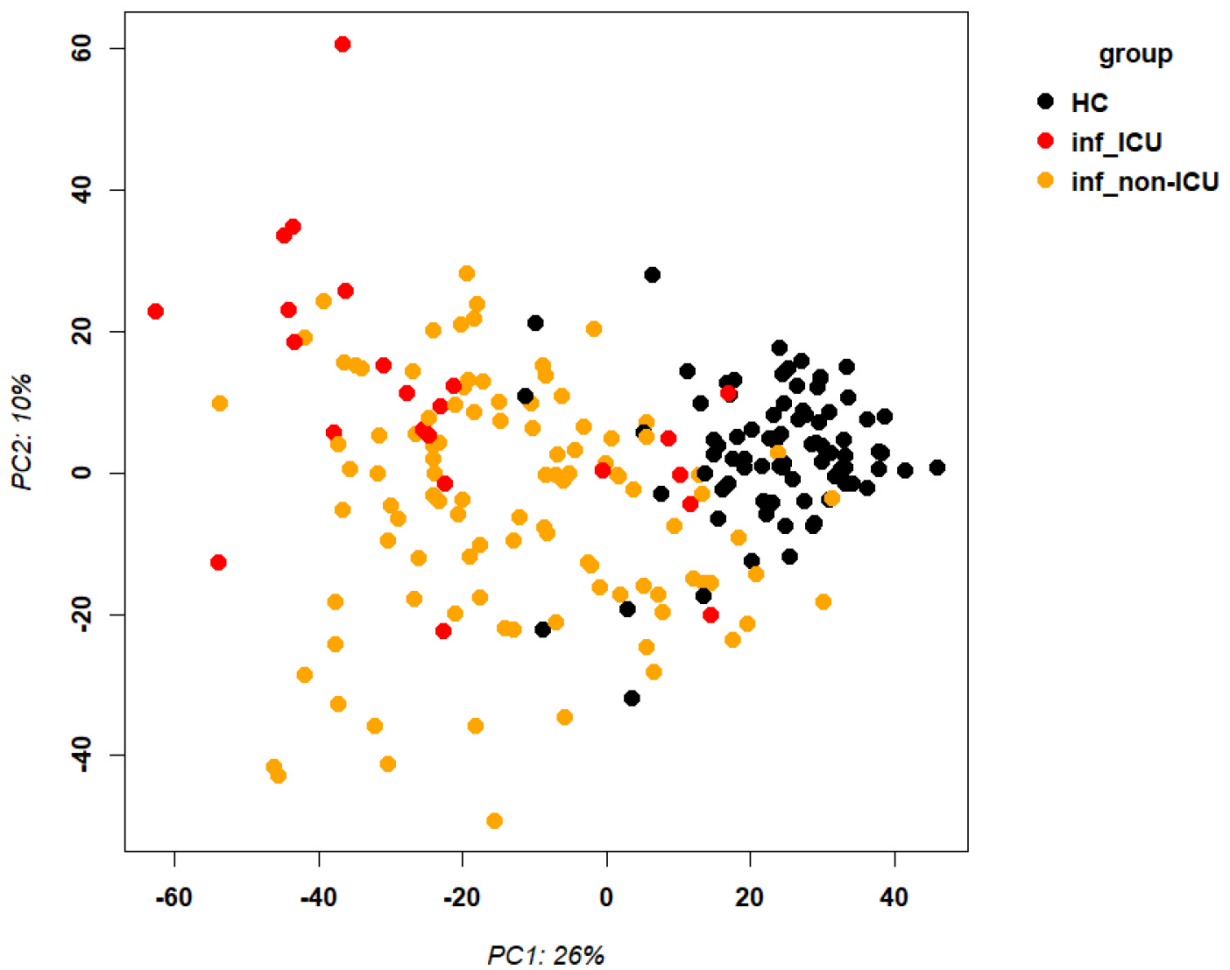
Principal component analysis of transcriptome expression. Principal component analysis plot for gene expression values of infected patients and healthy controls. Abbreviations: HC: healthy controls; inf_ICU infected patients at ICU; inf_non-ICU: infected patients not at ICU.

**Figure 2:**
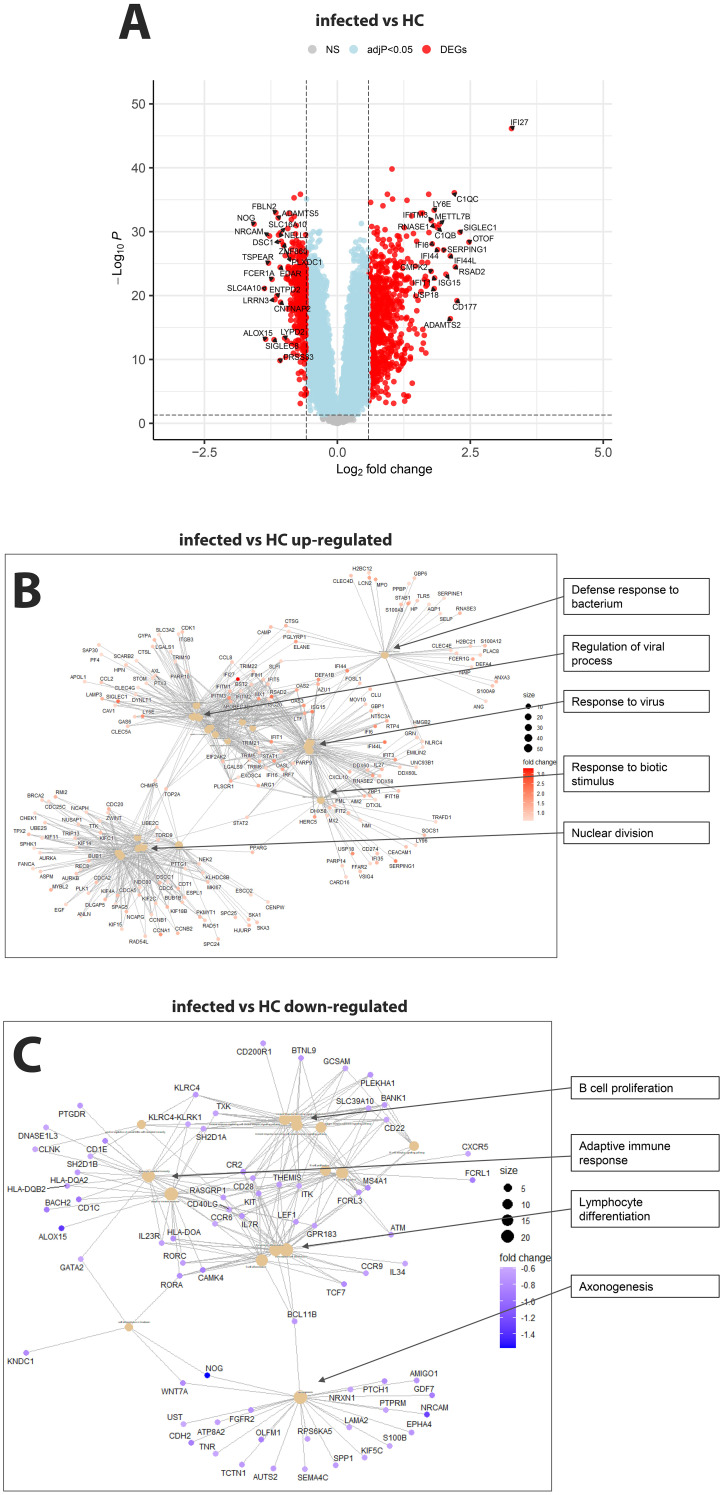
Comparison of infected patients versus healthy controls. **(A)** Volcano plot of infected patients versus healthy controls. y-axis: -log_10_ BH multiple testing adjusted p-values, x-axis: log_2_ fold change. DEGs are colored red, and the top 20 up- and down-regulated (by log-fold change) DEGs are labeled. Blue: not significant genes with an adjusted p-value < 0.05. Yellow: not significant genes with an absolute fold change of 1.5 (log_2_ = 0.5849625). Grey: NS, not significant. **(B)** Functional analysis using GO term enrichment for the up-regulated DEGs from the contrast of infected versus healthy controls. **(C)** Functional analysis using GO term enrichment for the down-regulated DEGs from the contrast of infected versus healthy controls.

RNA was isolated from the blood cells of 127 influenza-infected patients and 81 healthy controls and submitted to RNA sequencing. The samples that were used here for the analyses below are described in detail in **Data file 1** and their normalized expression values (unique per sample and patient, batch corrected) in **Data file 20**. Principal component analysis (PCA) demonstrated good separation between infected patients and healthy controls (**Figure 1**). Analysis of differentially expressed genes (DEGs) showed 701 upregulated and 367 downregulated genes when contrasting all infected patients versus healthy controls (**Figure 2A**; complete list of DEGs in **Data file 6**). The top 5 up- and down-regulated DEGs from this comparison and their known functions are listed in **Table 3**. Pathway analysis for DEGs between infected patients and healthy controls revealed that the top up-regulated genes were ‘Defense response to bacterium’, ‘Regulation of viral process’, ‘Response to virus’, ‘Response to biotic stimulus’, ‘Nuclear division’ (**Figure 2B**) and the top down-regulated genes were ‘B cell proliferation’, ‘Adaptive immune response’, ‘Lymphocyte differentiation’, and ‘Axonogenesis’ (**Figure 2C**).

**Table 3:** DEGs and their known functions.

Gene symbol	Identified as ..	Gene name	Known function
*IFI27*	DEG of infected versus healthy controls; up-regulated (Data file 6)	Interferon Alpha Inducible Protein 27	Encodes RNA polymerase II-specific DNA-binding transcription factor with lamin binding activity, predicted to be involved in defense response to other organism, protein K48-linked ubiquitination, and pyroptosis and acts upstream of or within negative regulation of transcription by RNA polymerase II and regulation of protein export from nucleus.
*OTOF*	DEG of infected versus healthy controls; up-regulated (Data file 6)	Otoferlin	Encodes protein with AP-2 adaptor complex binding activity and calcium ion binding activity, predicted to be involved in regulation of neurotransmitter secretion and synaptic vesicle priming.
*SIGLEC1*	DEG of infected versus healthy controls; up-regulated (Data file 6)	Sialic Acid Binding Ig Like Lectin 1	Encodes protein that enables virion binding activity, and is involved in negative regulation of type I interferon production.
*CD177*	DEG of infected versus healthy controls; up-regulated (Data file 6)	CD177 Molecule	Encodes protein with calcium-dependent binding activity, integrin binding activity, and protease binding activity, predicted to be involved in neutrophil extravasation, positive regulation of superoxide anion generation, and regulation of vesicle-mediated transport.
*RSAD2*	DEG of infected versus healthy controls; up-regulated (Data file 6)	Radical S-Adenosyl Methionine Domain Containing 2	Encodes protein with 4 iron, 4 sulfur cluster binding activity and protein self-association, predicted to be involved in defense response to virus, negative regulation of protein secretion, and negative regulation of viral genome replication.
*NOG*	DEG of infected versus healthy controls; down-regulated (Data file 6)	Noggin	Encodes protein with cytokine binding and protein homodimerization activity, predicted to be involved in embryonic morphogenesis, regionalization, and regulation of signal transduction.
*SLC4A10*	DEG of infected versus healthy controls; down-regulated (Data file 6)	Solute Carrier Family 4 Member 10	Encodes protein with sodium:bicarbonate symporter activity, predicted to be involved in bicarbonate transport, regulation of short-term neuronal synaptic plasticity, and visual perception, and to play a role in brain development, locomotory exploration behavior, and proton transmembrane transport.
*ALOX15*	DEG of infected versus healthy controls; down-regulated (Data file 6)	Arachidonate 15-Lipoxygenase	Encodes protein acting on single donors with incorporation of molecular oxygen, incorporation of two atoms of oxygen and phosphatidylinositol-4,5-bisphosphate binding activity, predicted to be involved in cellular response to interleukin-13, fatty acid metabolic process, and positive regulation of ERK1 and ERK2 cascade.
*TSPEAR*	DEG of infected versus healthy controls; down-regulated (Data file 6)	Thrombospondin Type Laminin G Domain And EAR Repeats	Encodes protein involved in regulation of Notch signaling pathway and tooth mineralization, located in cell surface and stereocilium.
*NRCAM*	DEG of infected versus healthy controls; down-regulated (Data file 6)	Neuronal Cell Adhesion Molecule	Encodes protein with ankyrin binding activity predicted to be involved in angiogenesis, central nervous system development, and clustering of voltage-gated sodium channels.
*MMP8*	DEG of ICU versus non-ICU; higher in ICU (Data file 9)	Matrix Metallopeptidase 8	Encodes protein with serine-type endopeptidase and tumor necrosis factor binding activity, predicted to be involved in endodermal cell differentiation, positive regulation of tumor necrosis factor production, and proteolysis. Biomarker of COVID-19, aortic aneurysm (multiple), arthritis (multiple), breast cancer, and female reproductive organ cancer (multiple).
*OLFM4*	DEG of ICU versus non-ICU; higher in ICU (Data file 9)	Olfactomedin 4	Encodes protein with cadherin binding and structural molecule activity, predicted to be involved in positive regulation of substrate adhesion-dependent cell spreading.
*ADAMTS2*	DEG of ICU versus non-ICU; higher in ICU (Data file 9)	ADAM Metallopeptidase With Thrombospondin Type 1 Motif 2	Encodes protein with metalloendopeptidase activity, predicted to be involved to be involved in extracellular matrix organization, acts on collagen fibril organization, protein processing, and spermatogenesis.
*ARG1*	DEG of ICU versus non-ICU; higher in ICU (Data file 9)	Arginase 1	Encodes protein with arginase activity and manganese ion binding activity, predicted to be involved in negative regulation of T cell proliferation, negative regulation of type II interferon-mediated signaling pathway, and positive regulation of neutrophil mediated killing of fungus.
*MAOA*	DEG of ICU versus non-ICU; higher in ICU (Data file 9)	Monoamine Oxidase A	Encodes protein with monoamine oxidase activity and primary amine oxidase activity, predicted to be involved in biogenic amine metabolic process, dopamine catabolic process and positive regulation of signal transduction.
*IL1R2*	DEG of ICU versus non-ICU; higher in ICU (Data file 9)	Interleukin 1 Receptor Type 2	Encodes protein with interleukin-1 binding activity and interleukin-1 receptor activity, predicted to be involved in immune response, acts in negative regulation of gene expression and negative regulation of interleukin-1-mediated signaling pathway.
*ZDHHC19*	DEG of ICU versus non-ICU; higher in ICU (Data file 9)	Zinc Finger DHHC-Type Palmitoyltransferase 19	Encodes protein with S-palmitoyltransferase activity, predicted to be involved in protein targeting to membrane.
*DEFA3*	DEG of ICU versus non-ICU; higher in ICU (Data file 9)	Defensin Alpha 3	Encodes protein involved in antimicrobial humoral immune response mediated by antimicrobial peptide, innate immune response in mucosa, and intracellular estrogen receptor signaling pathway.
*PCOLCE2*	DEG of ICU versus non-ICU; higher in ICU (Data file 9)	Procollagen C-Endopeptidase Enhancer 2	Encodes protein with heparin binding activity, and peptidase activator activity, predicted to be involved in cellular response to leukemia inhibitory factor.
*TPST1*	DEG of ICU versus non-ICU; higher in ICU (Data file 9)	Tyrosylprotein Sulfotransferase 1	Encodes protein with homodimerization activity and protein-tyrosine sulfotransferase activity, predicted to be involved in 3'-phosphoadenosine 5'-phosphosulfate metabolic process and inflammatory response.
*CCL2*	DEG of ICU versus non-ICU; higher in non-ICU (Data file 9)	C-C Motif Chemokine Ligand 2	Encodes protein with CCR2 chemokine receptor binding activity and chemokine activity, predicted to be involved in cellular response to cytokine stimulus, leukocyte chemotaxis, and regulation of apoptotic process.
*ZNF703*	DEG of ICU versus non-ICU; higher in non-ICU (Data file 9)	Zinc Finger Protein 703	Encodes protein with DNA-binding transcription factor binding activity and metal ion binding activity, predicted to be involved in cellular response to estradiol stimulus, positive regulation of mammary gland epithelial cell proliferation, and regulation of transforming growth factor beta receptor signaling pathway.
*CLEC4F*	DEG of ICU versus non-ICU; higher in non-ICU (Data file 9)	C-Type Lectin Domain Family 4 Member F	Encodes protein with galactose binding activity and glycolipid binding activity, predicted to be involved in endocytosis, and NK T cell activation.
*HLA-DQB1*	DEG of ICU versus non-ICU; higher in non-ICU (Data file 9)	Major Histocompatibility Complex, Class II, DQ Beta 1	Encodes protein with peptide antigen binding activity, protein antigen binding activity, and toxic substance binding activity, involved in T cell receptor signaling pathway, antigen processing and presentation of exogenous peptide antigen via MHC class II, and humoral immune response.
*CDKN1C*	DEG of ICU versus non-ICU; higher in non-ICU (Data file 9)	Cyclin Dependent Kinase Inhibitor 1C	Encodes protein with kinase inhibitor activity, predicted to be involved in negative regulation of epithelial cell proliferation, positive regulation of transforming growth factor beta receptor signaling pathway, and regulation of DNA-templated transcription.
*CCL8*	DEG of ICU versus non-ICU; higher in non-ICU (Data file 9)	C-C Motif Chemokine Ligand 8	Encodes protein with phospholipase activator activity and protein kinase activity, predicted to be involved in antimicrobial humoral immune response mediated by antimicrobial peptide, calcium ion transport, and intracellular calcium ion homeostasis.
*HLA-DQA1*	DEG of ICU versus non-ICU; higher in non-ICU (Data file 9)	Major Histocompatibility Complex, Class II, DQ Alpha 1	Encodes protein with MHC class II protein complex binding activity and peptide antigen binding activity, predicted to be involved in antigen processing and presentation of exogenous peptide antigen via MHC class II, peptide antigen assembly with MHC class II protein complex, and response to type II interferon, acting upstream of antigen processing and presentation of peptide antigen and positive regulation of T cell differentiation.
*NR4A1*	DEG of ICU versus non-ICU; higher in non-ICU (Data file 9)	Nuclear Receptor Subfamily 4 Group A Member 1	Encodes protein with DNA-binding transcription factor activity, RNA polymerase II-specific and sequence-specific double-stranded DNA binding activity, predicted to be involved in cellular response to vascular endothelial growth factor stimulus, endothelial cell migration, and positive regulation of endothelial cell proliferation.
*CYP4F22*	DEG of ICU versus non-ICU; higher in non-ICU (Data file 9)	Cytochrome P450 Family 4 Subfamily F Member 22	Encodes protein with monooxygenase activity, predicted to be involved in ceramide biosynthetic process.
*IL4I1*	DEG of ICU versus non-ICU; higher in non-ICU (Data file 9)	Interleukin 4 Induced 1	Encodes protein with L-amino-acid oxidase activity, predicted to be involved in aromatic amino acid family catabolic process, negative regulation of T cell mediated immune response to tumor cell, and regulation of T cell activation.

List of DEGs identified in different contrasts and their known functions. Information on gene symbols and names from Gene Cards (GeneCards), information on gene functions from (Alliance_of_Genome_Resources) and subsequently edited manually.

### Transcriptomes analysis of infected ICU versus infected non-ICU patients and to healthy controls

**Figure 3:**
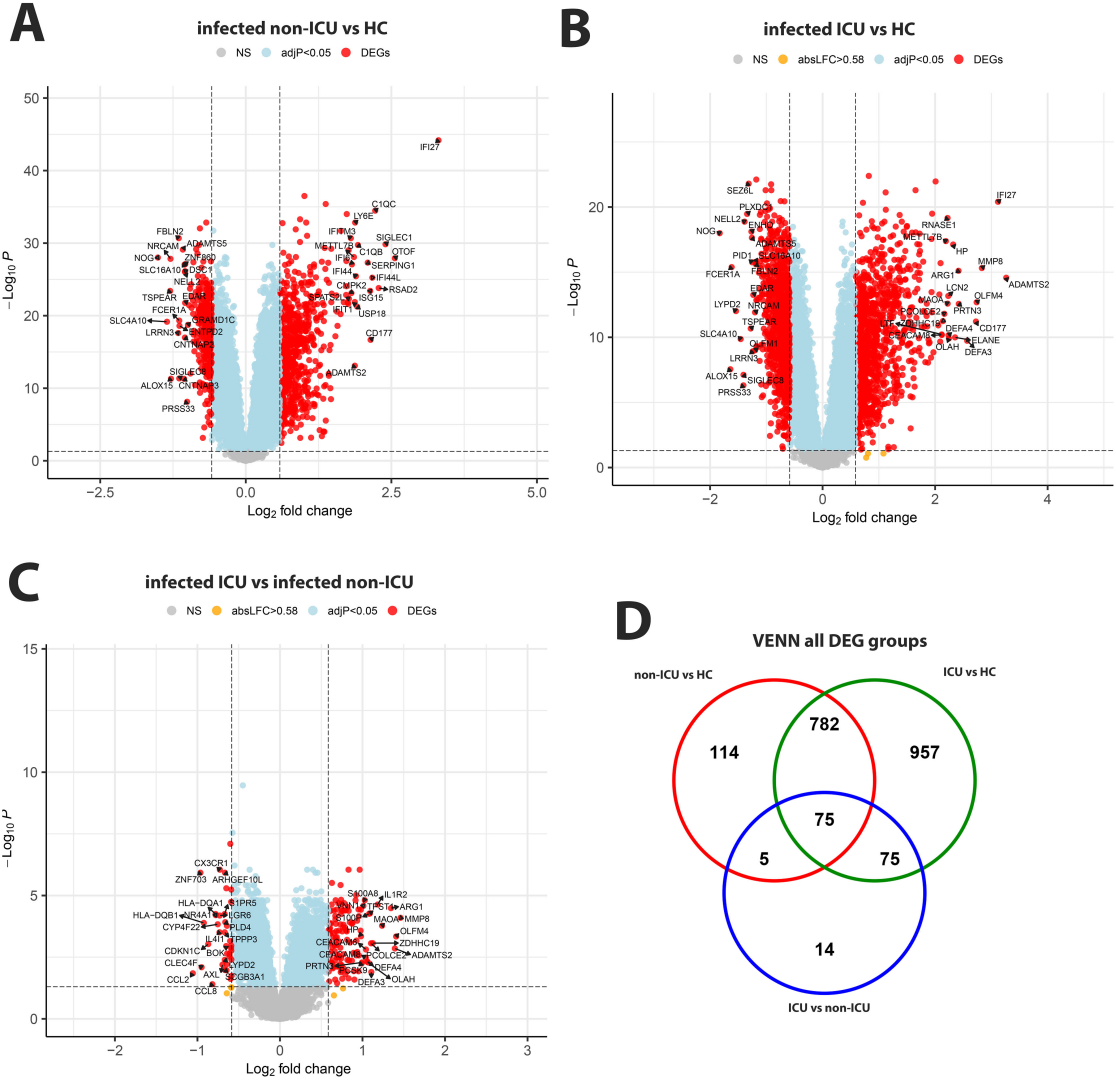
Comparison of infected non-ICU patients and ICU patients versus healthy controls, ICU versus non-ICU patients and corresponding VENN diagram. **(A)** Volcano plot of infected non-ICU patients versus healthy controls. **(B)** Volcano plot of infected ICU patients versus healthy controls. **(C)** Volcano plot of infected ICU patients versus infected non-ICU patients. See **Figure 2** for more details on volcano plots. **(D)** Venn diagram illustrating the overlaps between the DEGs from contrasts of ICU and non-ICU patients versus healthy controls and between ICU and non-ICU patients. A total of 2,022 DEGs were identified in all three groups (all genes combined), and 75 DEGs were commonly shared between the three groups (central overlap.

**Figure 4:**
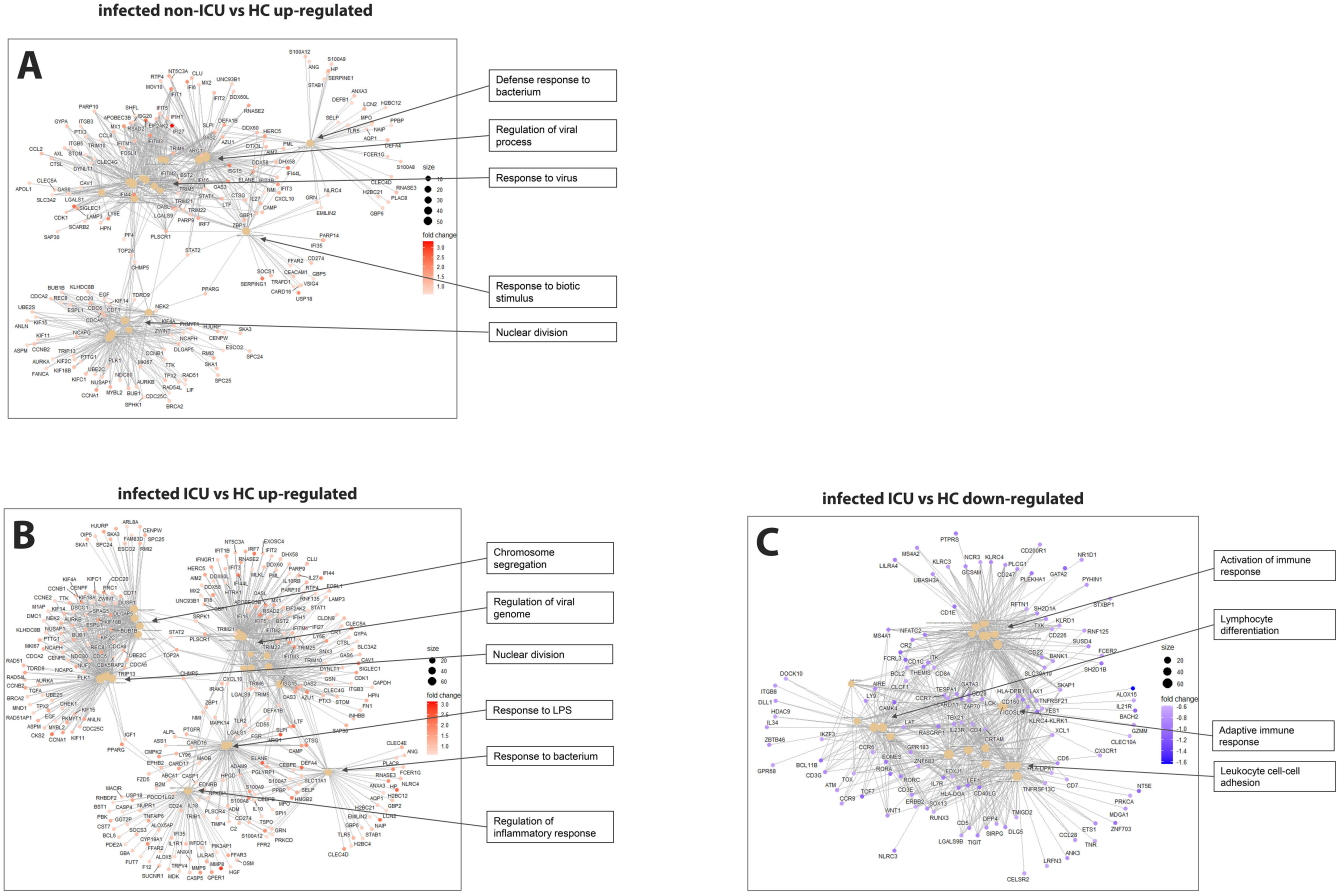
Pathway analysis of infected ICU and non-ICU patients versus healthy controls. **(A)** Functional analysis using GO term enrichment for the up-regulated DEGs from the contrast of infected non-ICU patients versus healthy controls. **(B)** Functional pathway analysis using GO term enrichment for the up-regulated DEGs from the contrast of infected ICU patients versus healthy controls. **(C)** Functional pathway analysis using GO term enrichment for the down-regulated DEGs from the contrast of infected ICU patients versus healthy controls.

**Figure 5:**
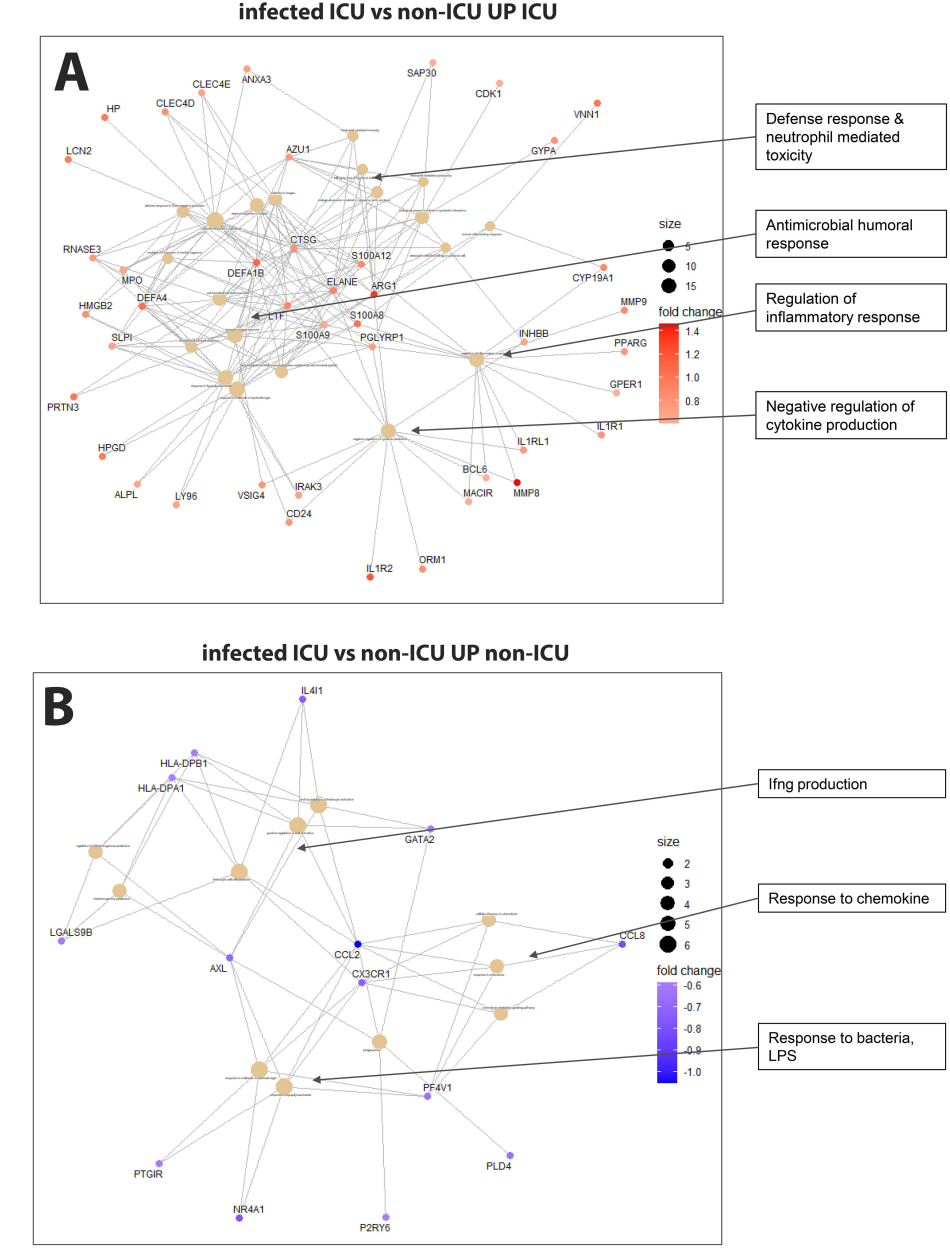
Pathway analysis of infected ICU versus non-ICU patients. **(A)** Functional analysis using GO term enrichment for the up-regulated DEGs (higher in ICU) from the contrast of infected ICU patients versus non-ICU patients. **(B)** Functional pathway analysis using GO term enrichment for the down-regulated DEGs (higher in non-ICU) from the contrast of infected ICU patients versus non-ICU patients.

Comparing non-ICU infected patients with health controls revealed 659 upregulated and 317 downregulated DEGs (**Figure 3A**) while ICU patients showed 1071 upregulated and 818 down-regulated DEGs when compared to healthy controls (**Figure 3B**). For the contrast of ICU patients and non-ICU patients, there were 132 up-regulated and 37 down-regulated DEGs (**Figure 3C**; complete lists of DEGs in **Data files 7–9**). **Table 3** lists, as examples, the top 10 DEGs from this comparison and their known functions. Of note, there was significant overlap between these groups with 857 DEGS shared between ICU and non-ICU patients when compared to healthy controls, and 75 DEGs shared between all contrasts (**Figure 3D**). For the DEGs of non-ICU patients compared to healthy controls, pathways for ‘Defense response to bacterium’, ‘Regulation of viral process’, ‘Response to virus’, ‘Response to biotic stimulus’, and ‘Nuclear division’ were up-regulated genes (**Figure 4A**), and no pathway associations were detected for the down-regulated genes. For DEGs of ICU patients compared to healthy controls, pathways for ‘Chromosome segregation’, ‘Regulation of viral genome’, ‘Nuclear division’, ‘Response to LPS’, ‘Response to bacterium’, ‘Regulation of inflammatory response’ were up-regulated genes (**Figure 4B**) and ‘Activation of immune response’, ‘Lymphocyte differentiation’, ‘Adaptive immune response’, and ‘Leukocyte cell-cell adhesion’ were down-regulated genes (**Figure 4C**). The direct comparison of ICU versus non-ICU revealed pathways for ‘Defense response & neutrophil-mediated toxicity’, ‘Antimicrobial humoral response’, ‘Regulation of inflammatory response’, ‘Negative regulation of cytokine production’ that were higher in ICU patients (**Figure 5A**) and pathways for ‘IFNG production’, ‘Response to chemokine’, ‘Response to bacterium’, and ‘Response to LPS’ that were higher in non-ICU patients (**Figure 5B**).

### Analysis of age and sex in driving transcriptome differences

In addition, we contrasted female versus male and young versus old patients, stratified by ICU and non-ICU. Only few DEGs were found in both comparisons. The results (**Supplementary Figure S1**) and discussion can be found in the supplementary material.

### Genotyping of participants

**Figure 6:**
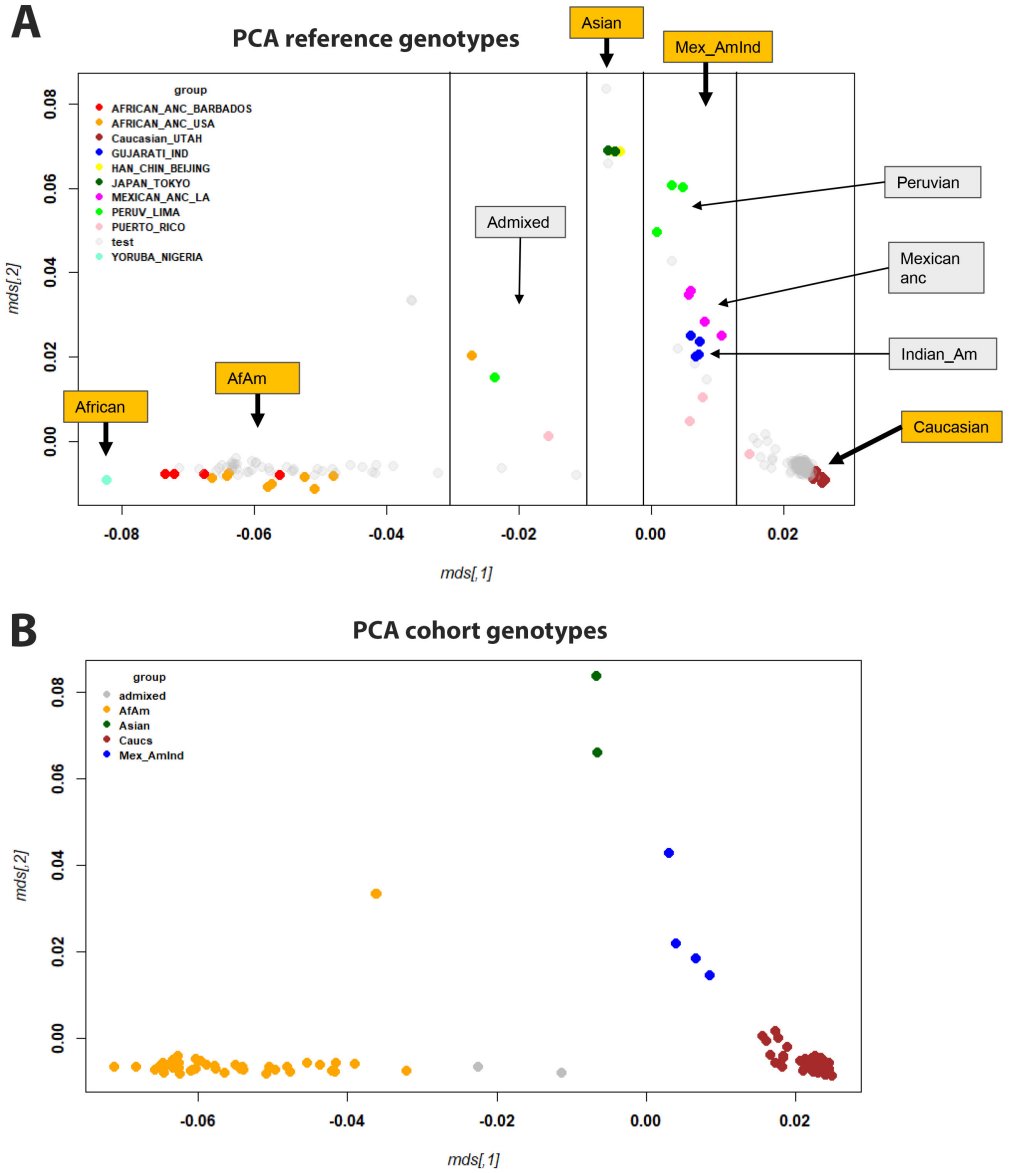
MDS plots of genotypes. **(A)** MDS plot showing all samples, with reference samples colored and participants in gray. **(B)** MDS plot of participants analyzed in this study (only a single representation for each participant, no reference genomes), colored for ethnicity. Abbreviations: African-American (AfAm), Mexican American Indians (Mex_AmInd), Mexican ancestry (Mexican anc), Indian American (Indian_Am).

Analysis of the genotype date after quality control revealed that the percent of missing SNPs per sample ranged from 2% to 6% (mean, 4%) with results differing somewhat between batches (means: SIG_geno_0718: mean = 2%; SIG_geno_0419: mean = 4%; SIG_geno_2020: mean = 5%; SIG_geno_2022: mean = 5%) (**Supplementary Figure S2A**). Only four SNPs were absent from all samples. The minor allele frequency ranged from 0 to 0.5 (**Supplementary Figure S2B**). These genotyping data and the inclusion of references to samples from the Coriell Institute HAPMAP collection allowed us to assign ethnicity to each patient from our cohort and to correlate genotype to gene expression data. The multidimensional scaling (MDS) plot of the reference genotypes showed a clear separation of ethnic groups (**Figure 6A**; colored for the ethnicity of the reference genomes). Based on the MDS components in **Figure 6A**, we assigned ethnicity to almost all participants from our cohort to four ancestral groups (**Figure 6B**): African-American (AfAm, n = 46), Asian (n = 2), Caucasian (Caucs, n = 169), Mexican American Indians (Mex_AmInd, n = 4). For some samples, no clear assignment was possible (referred to as admixed, n = 2).

### Associations between transcriptome and genotype

**Figure 7:**
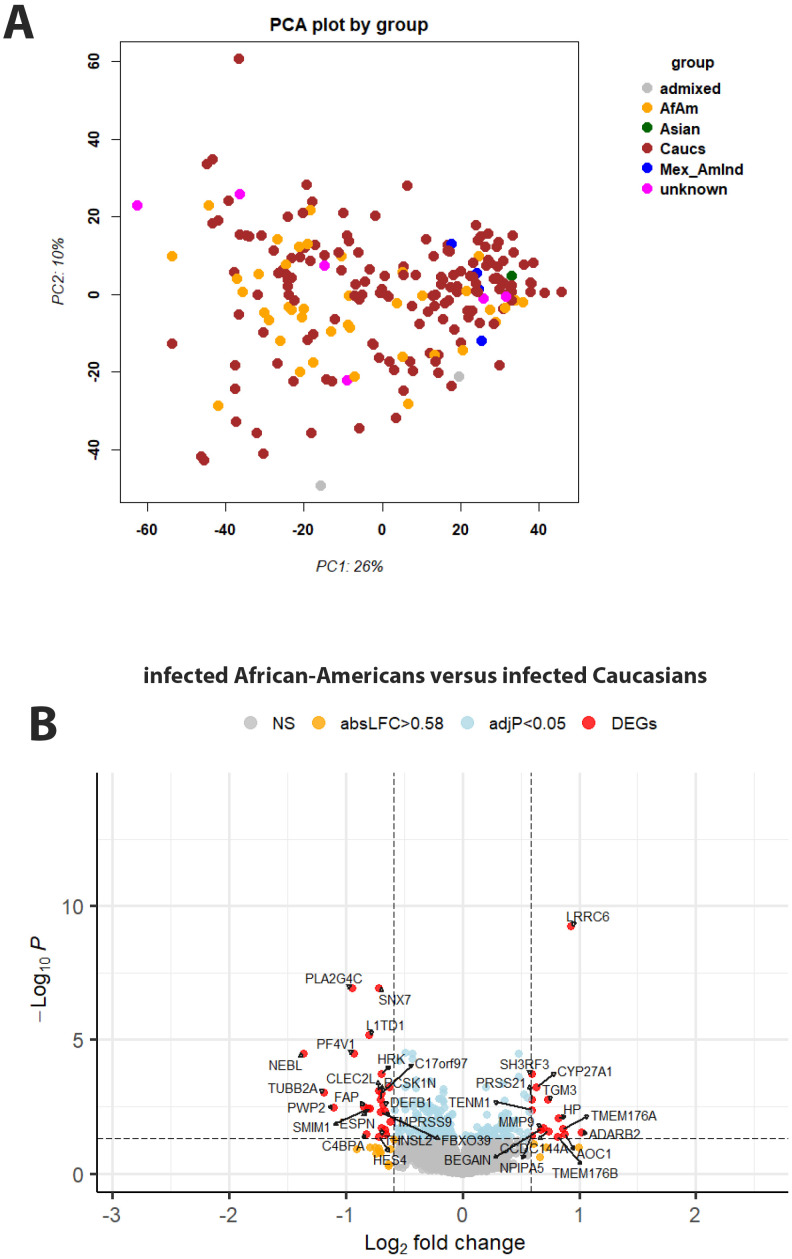
Principal component analysis and gene expression by genotype. **(A)** Principal component analysis plot for gene expression values of infected participants, colored by ethnicity. Abbreviations: African-American (AfAm), Caucasian (Caucs), Mexican American Indians (Mex_AmInd), Mexican ancestry (Mexican anc), Indian American (Indian_Am), no unambiguous assignment (admixed). **(B)** Volcano plot of DEGs for contrasts of infected Caucasian versus African-American patients. See **Figure 2** for more details on volcano plots.

After assigning ethnicity to every participant, we analyzed gene expression signals with respect to ethnicity (using **Data file 20** and sample descriptions from **Data file 1**). The PCA for normalized gene expression for all participants suggests PC1 and PC2 did not obviously separate by ethnicity (**Figure 7A**). We then specifically looked for genes in infected patients that were differentially expressed between African-Americans and Caucasians. Fifteen genes were expressed at significantly higher levels in infected African-Americans, and 24 were significantly higher in Caucasians (**Figure 7B**; **Data file 28**). No pathways were found to be associated with these genes. Six of the 39 DEGs overlapped with DEGs from infected versus healthy controls (*HP, MMP9, FBXO39, HES4, SMIM1*, and *FAP* (**Data file 6 and Data file 28**), indicating regulation after infection. All other ethnic groups were too small for a reasonable analysis.

**Figure 8:**
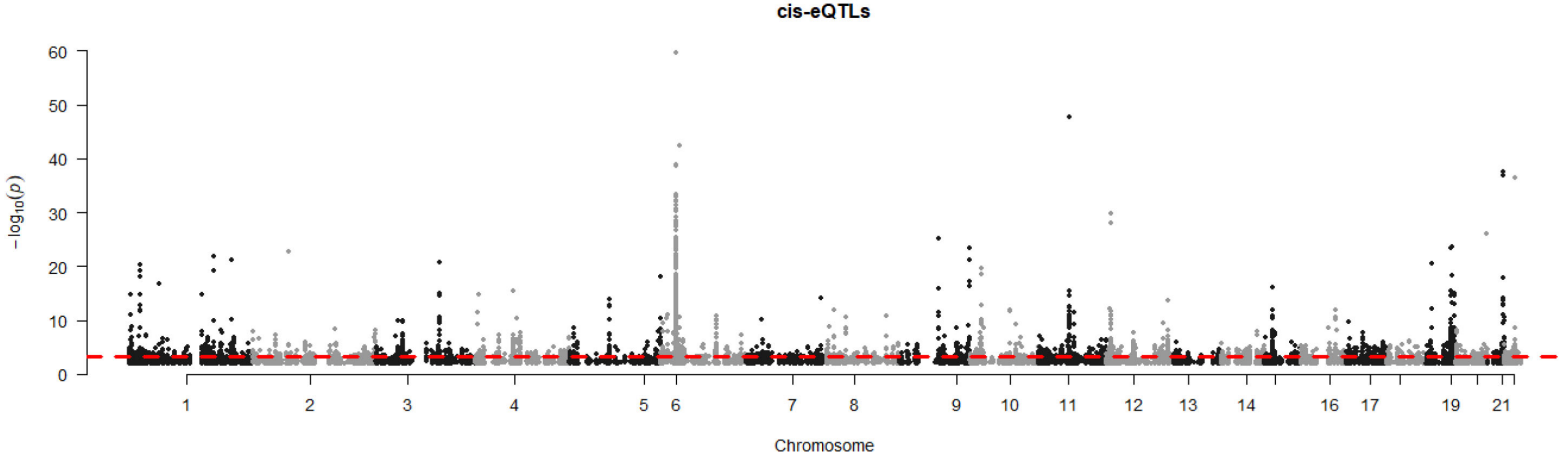
Manhattan plot of cis-eQTL analysis. Manhattan plot illustrating the results of cis-eQTL analysis for all 2,022 DEGs combined (DEGs from all comparisons presented in **Figures 2**, **3**). y-axis: -log10 of p-value, x-axis: genome position per chromosome.

**Figure 9:**
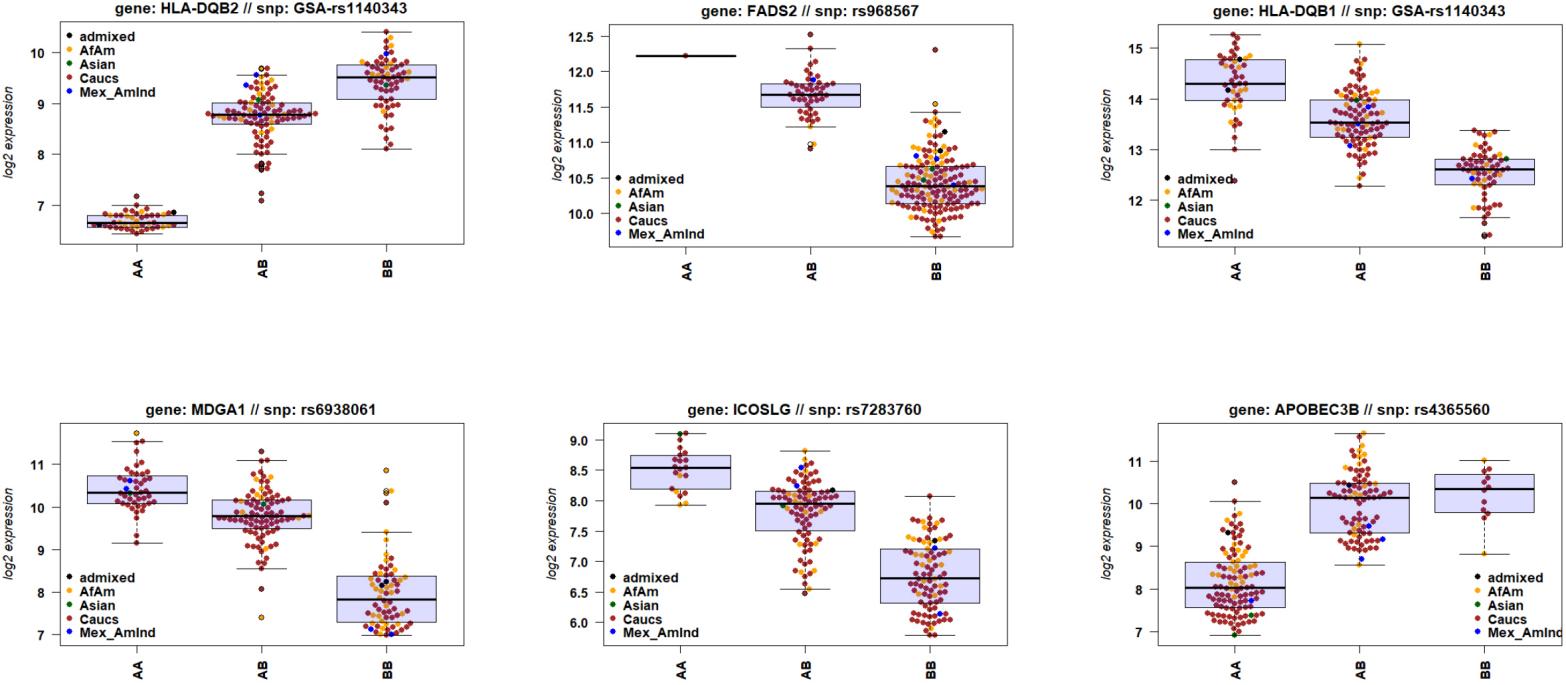
Gene expression levels of genes with a cis-eQTL. Boxplots of gene expression values of six top genes (by FDR) for e-QTL mapping, stratified by genotype. Box center line: median, box limits: upper and lower quartiles, whiskers: 1.5x interquartile range.

Also, our combined transcriptome and genotype data allowed us to identify genes for which the expression levels were correlated with genetic variations in or near the gene, so-called cis-eQTLs. Analyzing all DEGs combined (ICU versus healthy, non-ICU versus healthy, and ICU versus non-ICU), and including sex and ethnicity as covariates, showed that 871 DEGs were significantly correlated (adjusted *P* < 10^-5^, assuming an average of 100 SNPs per gene) with a genetic variant (**Data file 13**). The Manhattan plot showed the strongest cis-eQTLs on chromosome 6 (**Figure 8**) at the location of the HLA cluster. Sixty-one genes were mapped to chromosome 6, of which 16 were in the HLA region (30Mb – 34Mb). The top six most significant cis-eQTLs were *HLA-DQB2, FADS2, HLA-DQB1, MDGA1, ICOSLG*, and *APOBEC3B;* **Data file 13**). **Figure 9** illustrates their expression levels, stratified by genotype at its locus. In almost all cases, genotypes were distributed across ethnicities. Of note, almost all African-American patients (except one) carried genotype BB for *FADS2*.

Of the six DEGs from the comparison of infected African-American versus Caucasian patients, four DEGs (*SMIM1, FBXO39, HES4*, and *HP;* **Data file 28 and Data file 13**) also exhibited a significant cis-eQTL (**Supplementary Figure S3A**) stratified by ethnicity; **Supplementary Figure S3B** stratified by genotype; **Data file 14**). Gene *HP* had the lowest significance for a cis-eQTL and did not show a clear separation. For gene *FAP*, participants’ genotypes were all homozygous (AA), except for one heterozygote (AB).

## Discussion

Here, we analyzed the host response in human blood after infection with the influenza virus. This study is one of the largest cohorts to date using RNAseq technology and analyzing the influenza infection transcriptome response in humans (17–33, 57). Our study contained a total of 208 samples, where 127 were from infected patients, with 23 requiring intensive care. In addition, very unique to this study, we determined the genotype of participants, allowing us to correlate gene expression with ethnicity and genetic variation.

Pathway analysis for DEGs between infected patients and healthy controls agreed with other studies of influenza-infected patients regarding upregulation of common virus-host defense pathways (**Figure 2**; **Data file 6**) (17–33) and other respiratory virus infections (33). The main responses for up-regulated genes encompass interferon-stimulated genes and chemokine/cytokines. Down-regulated genes represent adaptive immune responses, most likely due to lymphopenia and suppression by up-regulated early inflammatory pathways, and most likely recruitment of adaptive immune cells into the lung.

Our results confirmed that *IFI27* (Interferon Alpha Inducible Protein 27; Volcano plot in **Figure 2A**; and list of DEGs in **Data file 6**) showed the strongest increase in influenza-infected individuals compared to healthy controls. *IFI27* is an interferon-induced gene involved in polymerase II-specific DNA-binding transcription factor binding activity and cellular apoptosis (29, 58) and is mainly expressed in dendritic cells (29). This study confirmed earlier observations describing its strong expression in the blood of infected influenza-infected patients (29, 59). In another study, we also observed top-level expression of *IFI27* for human metapneumovirus infections but not for enterovirus/rhinovirus infections (33). *IFI27* may, therefore, serve as a molecular biomarker in human blood to distinguish influenza and metapneumovirus from other respiratory infections. However, *IFI27* was not a distinguishing factor for ICU admission. More studies with different respiratory and non-respiratory viral, bacterial, and fungal infections will be required to confirm *IFI27* as robust diagnostic biomarker.

Pathways for inflammatory responses and neutrophil-mediated toxicity were higher in ICU patients, and pathways for IFNG production and response to chemokines were higher in non-ICU patients. These are similar observations as described in previous studies (25, 32, 59). The high activation of inflammatory responses, and especially the high activation of neutrophil responses, is the most likely cause of the observed immunopathology in ICU patients (25, 32). However, only some DEGs (169) were statistically significant in the direct contrast between ICU and non-ICU patients. This observation suggests that the magnitude of responses maybe different for many more genes but without being statistically significant (mostly higher in ICU patients).

It is worth noting that many of the DEGs from the comparison of ICU versus non-ICU patients (see **Data file 9** for list of DEGs) exhibit known functions in macrophages, suggesting that dysregulation of macrophages may also contribute to severe influenza disease. *CCL2* (C-C Motif Chemokine Ligand 2) was down-regulated in the ICU patients as was *CX3CR1* (C-X3-C Motif Chemokine Receptor 1), which is associated with obesity, a risk factor for severe influenza disease (60). *ARG1* (Arginase 1) was also up-regulated, and ARG1-expressing macrophages promote wound healing and dampen T cells (61–64). In addition, *ADAMTS2* (ADAM Metallopeptidase With Thrombospondin Type 1 Motif 2) was upregulated. *ADAMTS2* expression is associated with CD14 monocytes and alveolar macrophages, and it is known to function as a pro-collagen factor (65–67).

To our knowledge, this study is the first to use genotyping more precisely than using questionnaires and correlate ethnicity to gene expression differences. Genes influencing T cell signaling and co-stimulation (*ICOSLG, HLA-DQB2, and HLA-DQB1*) and type I interferon (*FADS2*, *MDGA1*, and *APOBEC3B*) were associated with genetic variation (Data file 13, **Figure 9**). *ICOSLG* (Inducible T Cell Costimulator Ligand) codes for a cell co-stimulator. Its loss leads to immunodeficiency (Roussel et al., 2018). *HLA-DQB2* (Major Histocompatibility Complex, Class II, DQ Beta 2) encodes a TCR signaling receptor (GeneCards). *FADS2* (Fatty Acid Desaturase 2) influences type I interferon response in CD4 cells (68). *MDGA1* (MAM Domain Containing Glycosylphosphatidylinositol Anchor 1) affects type I IFN in epithelial cells (69). *APOBEC3B* (Apolipoprotein B MRNA Editing Enzyme Catalytic Subunit 3B) affects type I IFN and was associated with COVID severity in African-Americans (70, 71). However, our study did not find ethnicity as a distinguishing factor, although it had higher expression in individuals of any ethnicity with AA or BB genotypes.

Only a few DEGs were different between severity groups for influenza-infected African-Americans and Caucasians, suggesting only a minor role in the risk of progressing to severe influenza. Four genes showed different expression between these two ethnic groups and also exhibited genetic variations in cis (within or close to the transcribed region of the genes), suggesting that differences in expression level are caused by genetic differences (**Supplementary Figure S3** and **Data file 14**). *SMIM1* (Small Integral Membrane Protein 1 (Vel Blood Group) encodes a protein that is the antigen for the Vel blood group, which participates in red blood cell formation. These proteins are part of SCF complexes, acting as protein-ubiquitin ligases. It does not have any known function in the host response to infections (GeneCards). *FBXO39* (F-Box Protein 39) is a member of the F-box protein family, containing an F-box motif. It is associated with Lymphogranuloma Venereum and Granuloma Inguinale disease. Thus, it may have a function in the host’s defense against infections (GeneCards). *HES4* (Hes Family BHLH Transcription Factor 4) is predicted to be part of the chromatin and to enable DNA-binding transcription factor activity and RNA polymerase II sequence-specific DNA binding activity. It does not have any known function in the host response to infections (GeneCards). The function of these genes in the immune defense against infections is unknown, except for *HP*. Thus, it is more likely that the few numbers of DEGs and their functions do not explain the observed differences in the population and that the observed differences in clinical outcomes could be due to socioeconomic differences or differences in access to care rather than biological factors (72).

Our study has several limitations. Although the cohort was large compared with other studies in the field, it only detected small differences when groups were stratified by severity plus sex and age. Thus, even larger group sizes may be needed to detect significant differences between these groups. Transcriptome analyses and genotype analyses were performed in several batches, which required batch corrections. Such corrections may result in a lower power to detect more subtle differences. We did not include patients younger than 18 years in our study, and thus could not analyze responses in children and adolescents. We did not perform a time course analysis for differential gene expression, because the number of patients from whom we collected multiple time data was too limited. Furthermore, the samples here were collected on ‘day 1’ of a patient’s presentation at the hospital or the first blood draw from ICU patients. Thus, the time of infection or onset of symptoms was unknown, which did not allow us to analyze the kinetics of the host response over time. Both point-in-time deviations of gene signatures and their causal relationships are unclear but should be the subject of future studies (73). Our cohort was large enough for the detection of cis-eQTL, although higher numbers would be preferable for detecting more. However, our cohort size was too small for the identification of trans-eQTLs variants in genes that affect the regulation of other genes distantly located in the genome. Nevertheless, our data will allow future studies to include the cis-eQTLs genotype information in their analyses. While the genes identified here and elsewhere may serve as biomarkers for a better diagnosis and/or predictors of severe disease, they still need to be clinically validated. However, even if verified, it is unclear how they might be used early enough to alter the course of disease.

## Data Availability

The datasets presented in this study can be found in online repositories. The names of the repository/repositories and accession number(s) can be found in the article/**Supplementary Material**.
