# Supplementary material for: Host response to influenza infections in human blood: association of influenza severity with host genetics and transcriptomic response
Source: Front Immunol. 2024 Aug 13;15:1385362. doi: 10.3389/fimmu.2024.1385362 (PMC11347429; doi:10.3389/fimmu.2024.1385362)
Supplement: Supplementary file 1 [file DataSheet1.docx]

# Supplementary material

## Comparison of female versus male patients

Comparing DEGs for males and females revealed no up-regulated DEGs in males for non-ICU patients or ICU patients, except for Y-chromosome-encoded genes. Only 2 up-regulated DEGs (*HEPH, MAP7D2*) were detected in females in both groups (Figure S1A, B; Data files 10, 11), both genes are X-linked.

## Comparison of old versus young patients

When comparing the transcriptome responses of old (≥65 years) versus younger patients (<65 years), only few DEGs (9 up- and 2 down-regulated) were detected (Figure S1C; Data file 12). Since interferons and interferon response genes are a major host defense mechanism against viral infections, we investigated whether the IFN response was a distinguishing factor. Our results showed that almost all interferon response genes (taken from the MSigDB database (INTERFERON_ALPHA_BETA_SIGNALING)) were expressed at a lower level in old compared to young patients (Figure S1D; Data files 29 and 30). However, these genes did not reach statistical significance in the direct comparison of old versus young.

# Discussion

Earlier studies described a difference in the immune responses of male and female patients (for review, see (Klein and Flanagan, 2016)). However, in our cohorts, we did not observe a statistically significant difference in gene expression between sexes, even when stratified by severity. The contrast of females versus males for all infected patients revealed the same DEGs with one exception: *UCHL1* was slightly higher in males (data not shown). An analysis of untreated and interferon-induced immune cell types in male and female mice identified 14 genes (*OAS3, RSAD2, SRSF2, ABCG1, SET, PHB2, KCNN4, INPP5F, KLF13, SEPHS2, AK007249, ABHD8, EIF2S3Y*) that were differentially regulated between sexes (Gal-Oz et al., 2019). None of these genes were differentially expressed between sexes in our study. One reason for not detecting sex-specific differences in our cohort may be that our patient cohort was too heterogeneous (by age, ethnicity, or unknown covariates) to allow for the detection of significant DEGs between sexes.

Influenza and SARS-CoV-2 infections are generally more severe in older patients compared to young patients (Statista;Feng et al., 2021b;Derqui et al., 2022). We, therefore, compared the blood responses in older (or equal to 65 years) versus young patients (18 to 65 years). Relatively few DEGs were found to be associated with age (9 up- and 2 down-regulated). The upregulated DEGs included *CA1*, *FAM83A*, *CHIT1*, *HP*, *RETN, SERPINB10*, *TMEM52B*, *KLHDC8A*, and *GLDN*, and down-regulated DEGs included *LRRN3* and *CD248.* Of these DEGs, five have a known function in infection/inflammation processes (information taken from (GeneCards)): *CHIT1* (Chitinase 1) protein is secreted by activated human macrophages and may play a role in the degradation of chitin-containing pathogens (GeneCards). *HP* (Haptoglobin) encodes a protein of the acute phase response that binds free hemoglobin to prevent it from providing nutrients to pathogens and for degradative enzymes to gain access and prevent the loss of iron through the kidneys. The release of haptoglobin is regulated by the liver, and often activated by cytokines of the interleukin 1 family during the onset of infection (GeneCards;Ayari et al., 2020). In addition, the encoded protein exhibits antimicrobial activity against bacteria. HP was also stronger up-regulated in the contrast of African-Americans versus Caucasians (higher in infected Caucasians). *RETN* (Resistin) also encodes a protein with antimicrobial functions against both Gram-positive and Gram-negative bacteria with related pathways associated with the innate immune response. Identification of these is interesting given the exacerbated susceptibility to bacteria during influenza infection (GeneCards;Smith and McCullers, 2014;Pine et al., 2018;Feng et al., 2021a;Perpiñan et al., 2022). *SERPINB10* (Serpin Family B Member 10) encodes a serpin peptidase inhibitor controlling the regulation of protease functions during hematopoiesis (GeneCards). It is also activated after vaccination in stimulated PBMCs (Quach et al., 2023). *CD248* (CD248 molecule) is involved in extracellular matrix binding activity and cell migration, lymph node development, and the regulation of endothelial cell apoptosis (GeneCards). These genes may be involved in causing or being associated with severe influenza disease in older patients. A limitation of this study is that patient deaths were not recorded, so our analysis could not detect DEGs related to high mortality. Furthermore, we observed that interferon response genes were reduced in the elderly compared to young patients, which may be one reason for the higher risk in the elderly for severe infections. Autoantibodies against interferons were found to be associated with severe influenza pneumonia (Zhang et al., 2022). Also, for SARS-CoV-2 infections, higher susceptibility was linked to suppression of the interferon pathway due to autoantibodies (Feng et al., 2021b).

# Figure Legends

## Figure S1. DEGs for contrasts of females versus males and effect of age.

**(A)** Volcano plot female versus male patients in non-ICU patients. **(B)** Volcano plot female versus male patients in ICU patients. **(C)** Volcano plot old (older or equal to 65 years) versus young patients for all infected patients. y-axis: -log_10_ BH multiple testing adjusted p-values, x-axis: log_2_ fold change. DEGs are colored red, and the top 20 up- and down-regulated (by log-fold change) DEGs are labeled. Blue: not significant genes with an adjusted p-value < 0.05. Yellow: not significant genes with an absolute fold change of 1.5 (log_2_ = 0.5849625), Grey: NS, not significant. **(D)** Barplot of interferon-signaling genes for old and young patients. Values per gene represent the log_2_-fold change for each group compared to healthy controls.

## Figure S2. Percent missing SNPs and minor allele frequency.

**(A)** Histogram showing the percent of missing SNPs for each sample. Note that samples were analyzed in four batches. **(B)** Histogram showing the frequency distribution of minor allele frequencies for all SNPs.

## Figure S3. Gene expression levels of individual genes with a cis-eQTL by genotype.

**(A)** Boxplots of gene expression values of top six (by FDR) DEGs from the comparison of infected African-American versus Caucasian patients, stratified by ethnicity. **(B)** Boxplots of gene expression values of four DEGs from a comparison of infected African-American versus Caucasian patients, stratified by genotype. The y-axis shows normalized log_2_ expression levels. Box center line: median, box limits: upper and lower quartiles, whiskers: 1.5x interquartile range.

# References

Ayari, A., Rosa-Calatrava, M., Lancel, S., Barthelemy, J., Pizzorno, A., Mayeuf-Louchart, A., Baron, M., Hot, D., Deruyter, L., Soulard, D., Julien, T., Faveeuw, C., Molendi-Coste, O., Dombrowicz, D., Sedano, L., Sencio, V., Le Goffic, R., Trottein, F., and Wolowczuk, I. (2020). Influenza infection rewires energy metabolism and induces browning features in adipose cells and tissues. *Commun Biol* 3**,** 237.

Derqui, N., Nealon, J., Mira-Iglesias, A., Díez-Domingo, J., Mahé, C., and Chaves, S.S. (2022). Predictors of influenza severity among hospitalized adults with laboratory confirmed influenza: Analysis of nine influenza seasons from the Valencia region, Spain. *Influenza Other Respir Viruses* 16**,** 862-872.

Feng, C.M., Cheng, J.Y., Xu, Z., Liu, H.Y., Xu, D.X., Fu, L., and Zhao, H. (2021a). Associations of Serum Resistin With the Severity and Prognosis in Patients With Community-Acquired Pneumonia. *Front Immunol* 12**,** 703515.

Feng, E., Balint, E., Poznanski, S.M., Ashkar, A.A., and Loeb, M. (2021b). Aging and Interferons: Impacts on Inflammation and Viral Disease Outcomes. *Cells* 10.

Gal-Oz, S.T., Maier, B., Yoshida, H., Seddu, K., Elbaz, N., Czysz, C., Zuk, O., Stranger, B.E., Ner-Gaon, H., and Shay, T. (2019). ImmGen report: sexual dimorphism in the immune system transcriptome. *Nat Commun* 10**,** 4295.

Genecards [*https://www.genecards.org/*](https://www.genecards.org/).

Interferon_Alpha_Beta_Signaling, M. REACTOME_INTERFERON_ALPHA_BETA_SIGNALING. [*https://www.gsea-msigdb.org/gsea/msigdb/human/geneset/REACTOME_INTERFERON_ALPHA_BETA_SIGNALING*](https://www.gsea-msigdb.org/gsea/msigdb/human/geneset/REACTOME_INTERFERON_ALPHA_BETA_SIGNALING).

Klein, S.L., and Flanagan, K.L. (2016). Sex differences in immune responses. *Nat Rev Immunol* 16**,** 626-638.

Perpiñan, C., Bertran, L., Terra, X., Aguilar, C., Binetti, J., Lopez-Dupla, M., Rull, A., Reverté, L., Yeregui, E., Gómez-Bertomeu, F., Peraire, J., Auguet, T., and On Behalf of Covid-Study, G. (2022). Resistin and IL-15 as Predictors of Invasive Mechanical Ventilation in COVID-19 Pneumonia Irrespective of the Presence of Obesity and Metabolic Syndrome. *J Pers Med* 12.

Pine, G.M., Batugedara, H.M., and Nair, M.G. (2018). Here, there and everywhere: Resistin-like molecules in infection, inflammation, and metabolic disorders. *Cytokine* 110**,** 442-451.

Quach, H.Q., Goergen, K.M., Grill, D.E., Haralambieva, I.H., Ovsyannikova, I.G., Poland, G.A., and Kennedy, R.B. (2023). Virus-specific and shared gene expression signatures in immune cells after vaccination in response to influenza and vaccinia stimulation. *Front Immunol* 14**,** 1168784.

Smith, A.M., and Mccullers, J.A. (2014). Secondary bacterial infections in influenza virus infection pathogenesis. *Curr Top Microbiol Immunol* 385**,** 327-356.

Statista influenza-us-mortality-rate-by-age-group. [*https://www.statista.com/statistics/1127799/influenza-us-mortality-rate-by-age-group/*](https://www.statista.com/statistics/1127799/influenza-us-mortality-rate-by-age-group/).

Zhang, Q., Pizzorno, A., Miorin, L., Bastard, P., Gervais, A., Le Voyer, T., Bizien, L., Manry, J., Rosain, J., Philippot, Q., Goavec, K., Padey, B., Cupic, A., Laurent, E., Saker, K., Vanker, M., Särekannu, K., García-Salum, T., Ferres, M., Le Corre, N., Sánchez-Céspedes, J., Balsera-Manzanero, M., Carratala, J., Retamar-Gentil, P., Abelenda-Alonso, G., Valiente, A., Tiberghien, P., Zins, M., Debette, S., Meyts, I., Haerynck, F., Castagnoli, R., Notarangelo, L.D., Gonzalez-Granado, L.I., Dominguez-Pinilla, N., Andreakos, E., Triantafyllia, V., Rodríguez-Gallego, C., Solé-Violán, J., Ruiz-Hernandez, J.J., Rodríguez De Castro, F., Ferreres, J., Briones, M., Wauters, J., Vanderbeke, L., Feys, S., Kuo, C.Y., Lei, W.T., Ku, C.L., Tal, G., Etzioni, A., Hanna, S., Fournet, T., Casalegno, J.S., Queromes, G., Argaud, L., Javouhey, E., Rosa-Calatrava, M., Cordero, E., Aydillo, T., Medina, R.A., Kisand, K., Puel, A., Jouanguy, E., Abel, L., Cobat, A., Trouillet-Assant, S., García-Sastre, A., and Casanova, J.L. (2022). Autoantibodies against type I IFNs in patients with critical influenza pneumonia. *J Exp Med* 219.
