# Supplementary figures and images for: Host response to influenza infections in human blood: association of influenza severity with host genetics and transcriptomic response
Source: Front Immunol. 2024 Aug 13;15:1385362. doi: 10.3389/fimmu.2024.1385362 (PMC11347429; doi:10.3389/fimmu.2024.1385362)

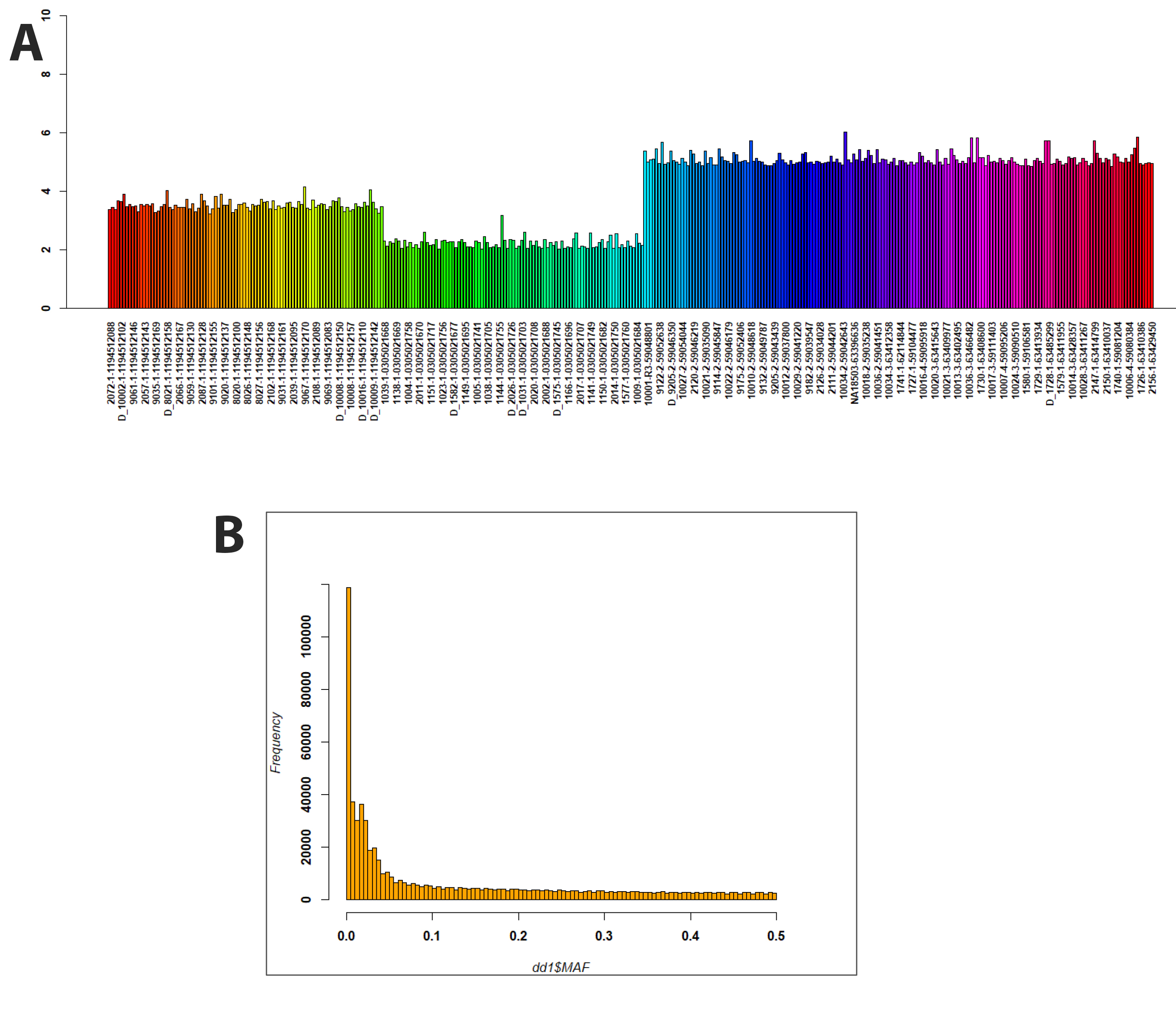

Supplement: Supplementary file 3 [file Image2.jpeg]

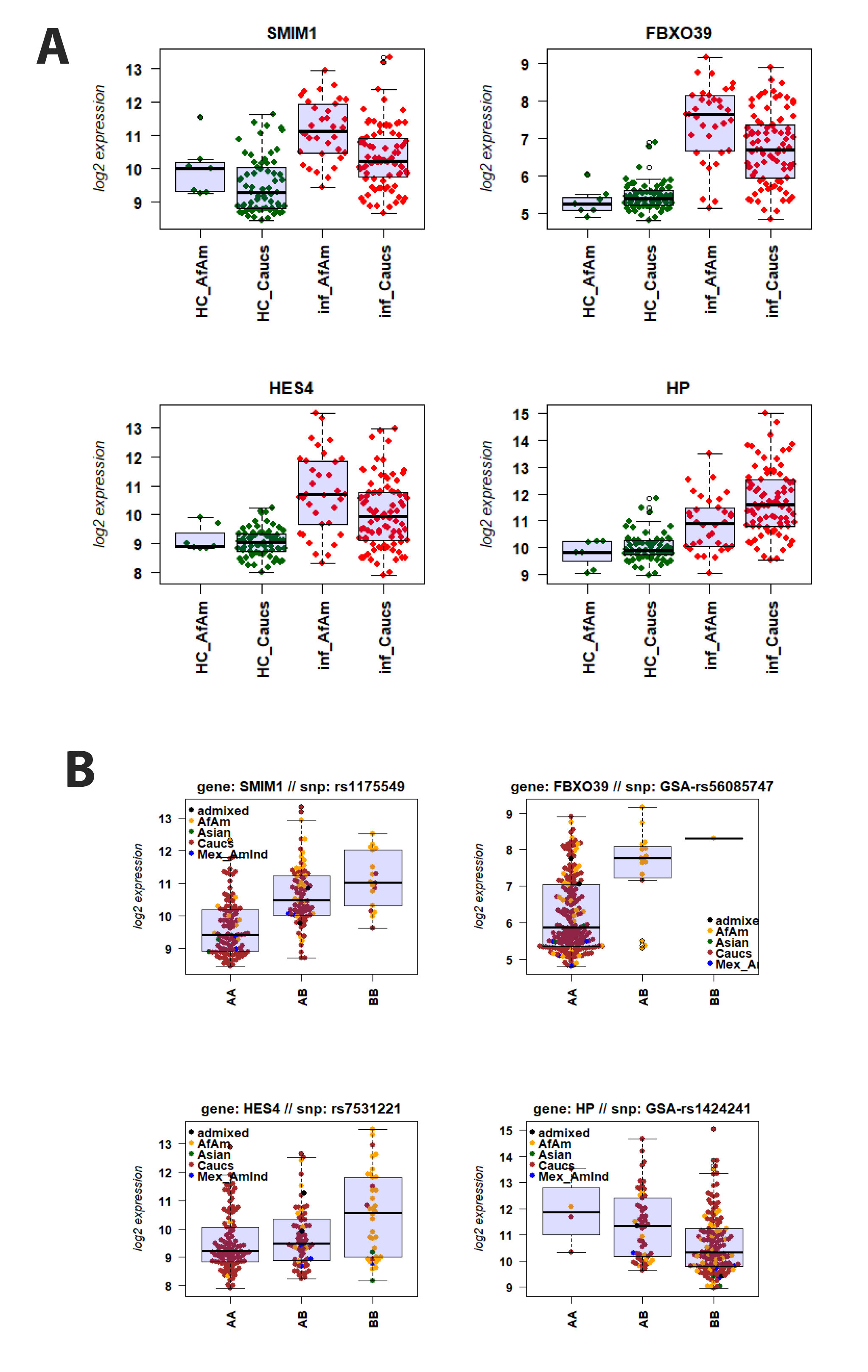

Supplement: Supplementary file 4 [file Image3.jpeg]
